# Influence of Engine Electronic Management Fault Simulation on Vehicle Operation

**DOI:** 10.3390/s22052054

**Published:** 2022-03-07

**Authors:** Branislav Šarkan, Michal Loman, František Synák, Michal Richtář, Mirosław Gidlewski

**Affiliations:** 1 Department of Road and Urban Transport, University of Žilina, 01026 Žilina, Slovakia; loman@stud.uniza.sk (M.L.); frantisek.synak@fpedas.uniza.sk (F.S.); 2 Department of Automotive Engineering and Transport, Kielce University of Technology, 25-314 Kielce, Poland; 3 Faculty of Mechanical Engineering, Technical University of Ostrava, 70800 Ostrava, Czech Republic; michal.richtar@vsb.cz; 4 Institute of Vehicles and Transportation, Military University of Technology (WAT), gen. Sylwestra Kaliskiego 2 Street, 00-908 Warsaw, Poland; miroslaw.gidlewski@wat.edu.pl; 5 Łukasiewicz Research Network—Automotive Industry Institute (Łukasiewicz-PIMOT), Jagiellońska 55 Street, 03-301 Warsaw, Poland

**Keywords:** engine power, emissions, air pollution, electrical management, fault codes, emission inspection, vehicle safety

## Abstract

The preparation of the fuel mixture of a conventional internal combustion engine is currently controlled exclusively electronically. In order for the electrical management of an internal combustion engine to function properly, it is necessary that all its electronic components work flawlessly and fulfill their role. Failure of these electronic components can cause incorrect fuel mixture preparation and also affect driving safety. Due to the effect of individual failures, it has a negative impact on road safety and also negatively affects other participants. The task of the research is to investigate the effect of the failure of electronic engine components on the selected operating characteristics of a vehicle. The purpose of this article is to specify the extent to which a failure of an electronic engine component may affect the operation of a road vehicle. Eight failures of electronic systems (sensors and actuators) were simulated on a specific vehicle, with a petrol internal combustion engine. Measurements were performed in laboratory conditions, the purpose of which was to quantify the change in the operating characteristics of the vehicle between the faulty and fault-free state. The vehicle performance parameters and the production of selected exhaust emission components were determined for selected vehicle operating characteristics. The results show that in the normal operation of vehicles, there are situations where a failure in the electronic system of the engine has a significant impact on its operating characteristics and, at the same time, some of these failures are not identifiable by the vehicle operator. The findings of the publication can be used in the drafting of legislation, in the field of production and operation of road vehicles, and also in the mathematical modeling of the production of gaseous emissions by road transport.

## 1. Introduction

Climate change is a key challenge facing the world today [1]. One of the main reasons for climate change is attributed to pollution. This pollution also causes serious public health problems in most parts of the world [2]. There are many sources of air pollution, such as car exhaust fumes, industrial exhaust fumes, waste incineration, etc. Places where the main source of air pollution is exhaust fumes from cars are very dangerous for the health of the population. Caban’s research has shown that such places are areas of congestion and various crossroads, especially in cities [3]. Studies show that air pollution causes many diseases, which lead to additional costs. These include costs associated with health care and reduced productivity [4,5].

At present, transport is one of the largest producers of CO2 emissions. Of all modes of transport, the largest producers are air transport, 13.4%, rail, 0.5%, water, 13.6%, others, 0.5%, road transport, 72%. Up to 60.7% are emissions from passenger cars. This is one of the main reasons why the topic is topical. There is currently a lot of pressure on vehicle manufacturers to reduce these emissions [6].

Harrison et al., in their study, examined the effectiveness of road vehicle emission controls in Europe and their implications for public health. Analysis of air quality measurement data from the United Kingdom and France have shown that road transport exhaust gases have a much greater impact on nitrogen dioxide concentrations than PM_2.5_. PM_2.5_ (particulate matter) and particulate matter concentrations have been declining significantly since 2011, due to the use of particulate filters in diesel engines, but little change has been observed between 1995 and 2015 in the area of nitrogen dioxide [7].

Emission regulation has become a very important part of today’s car production [8,9]. Manufacturers are trying to design vehicles that produce the lowest possible emissions during operation [10,11,12]. The introduction of increasingly stringent emission standards is significantly increasing the use of electronics in vehicles. The task of electric fuel preparation is to optimize the combustion of the engine, to such an extent that it produces the lowest possible quantity of emissions [13]. Mahdina et al. proved this in their research. They proved that a vehicle that uses electronic components in its operation achieves a lower quantity of emissions than a vehicle that works without them. They also examined fuel consumption, which was also lower compared to manually operated vehicles [14].

OBD (On-Board Diagnostics) is one of the important tools used to control emissions in today’s cars [15,16]. If the system does not match the selected OBD component, it generates data trouble codes (DTCs). Devices are available on the market that are able to read fault codes from the vehicle fault memory. In their output, they provide technicians with information about the damaged component [17,18].

The driver can be informed of a fault in one of the electronic components of the engine management by means of the MIL (malfunction indicator lamp) warning light, which lights up on the vehicle’s instrument panel when the so-called OBD threshold limits. During vehicle operation, there are situations when a fault stored in the ECU (engine control unit) memory does not cause the MIL to illuminate [19,20].

Emission inspections are carried out to prevent the movement of vehicles whose emissions exceed a set limit. The task of the emission inspection is to assess the condition of the vehicle brought to the workplace at regular intervals. The result of this inspection tells you whether the vehicle passes but is not OK to operate in real traffic. The operator must demonstrate that the vehicle meets the legal limits for exhaust emissions. However, there is considerable evidence that many vehicles operating on the road would not meet the relevant emission standards. The main reason is insufficient vehicle maintenance; as long as the engine settings do not meet the manufacturers’ specifications, it is not surprising that their performance and emissions are deteriorating [21].

The technical conditions of vehicles can also be affected by age. Sales of new vehicles in the European Union in 2021 have decreased by 25%, in comparison with the previous year, and the average age of the vehicles is 11.5 years [22,23]. Lower sales of new vehicles and the higher age of the used ones premise worse technical conditions of vehicles [24,25]. The emission inspection is usually performed every two years. Between two emission inspections, the vehicle may malfunction, resulting in an increase in some of the harmful components of the exhaust gases. The increase in one or more harmful components of the exhaust gases may be such that the vehicle is still evaluated as “OK”, but the concentration of these components is increased compared to the fault-free condition. In this case, even the implementation of emission control does not mean the cessation of excessive production of pollutants and, thus, deterioration of quality, especially in densely populated areas and with appropriate impacts. The operation of a vehicle with a breakdown leads both to increased emissions and often to a gradual worsening of the fault and its manifestations. An unresolved error can, thus, result in a significant deterioration of the technical condition of the vehicle and further deterioration of the consequences. The driver does not have to be informed of this error and of the excessive production of pollutants. If the driver is informed of the fault by the tell-tale signs but the fault is not accompanied by a significant reduction in engine power, it is not automatic for the fault to be rectified.

The research was a vehicle with a petrol engine with improved combustion. This means that emissions were measured at idle, as well as at increased engine speeds [26]. However, we encounter different theories regarding vehicle emissions. Kuranc, Zheng et al., in their research, describe that the emissions determined on the basis of the driving cycle used in vehicle type-approval (NEDC—New European Driving Cycle) do not correspond to reality. This is due to the fact that the vehicle’s engine is at a higher load than in the test during real operation. This means that both emission production and, thus, fuel consumption, will be higher than with type approval [27,28].

Engine power is one of the pieces of information that is commonly available data [29,30]. When measuring power on a roller dynamometer, it distinguishes between several types of power:

1. Engine power: measured on the power cylinder brake without any regard to the applicable world standards (ISO, DIN, EWG),
2. Corrected power: measured on the power cylinder brake and, at the same time, recalculated using the calculations of the applicable world standards (ISO, DIN, EWG). The corrected power is that of the engine, as stated by the manufacturer. It is a power that is affected by external influences, such as temperature, humidity, and air pressure.
3. Power on wheels: this is the power that the vehicle achieves on wheels.
4. Power dissipation: this is the value that arose as the sum of power losses from all passive/rotating resistors. These include losses on wheels, losses arising from the engagement of gears, differentials, bearings, and others [31,32].

The research examined the extent to which the failure of a selected component affects engine performance. This, together with the exhaust emissions, was measured at each electronic component failure simulation. Engine power can be considered part of the active safety of a vehicle. A sudden decrease in engine power can cause an accident, for example, in the event of an overtaking maneuver. A reduction in engine power may be the result of a new fault or an existing fault that has not been resolved for a long time. One of the components on which the fault simulation was performed was the crankshaft position sensor. Rizzoni et al., in their study, described several options for monitoring the crankshaft position sensor signal and the possibility of assessing the effect on engine function, in the event of a sensor failure [33].

The research demonstrates the extent to which a vehicle with selected electronic fuel preparation failures is operational. In addition to the measurement, the driver’s awareness of the failure of the selected electronic component will be monitored, i.e., whether the driver is aware of the failure of their vehicle. Vehicle emissions have a negative impact on the environment. Therefore, carrying out emission inspections is considered an integral part of our research. The benefit and uniqueness of this publication also lie in the complexity and volume of measurements performed. Comparable publications usually focus on only one component or part, and emissions, engine power, and driver information are not examined at the same time. For example, in publication [34], attention is focused only on the consequences of throttle position sensor failure in the area of emissions. The effect of the fault on the engine power has not been investigated in this case. The publication of [35] examined the effect of an electrical component failure on performance and emissions, but in this case, it was also only a single component, the oxygen sensor. The advantage of the complexity of the measurements reported in this publication and its benefit is also evident when compared to the measurements reported in the publications [36,37,38,39,40].

The selection of the vehicle used for measurements also contributes to the contribution of the article. A vehicle with a petrol engine with electronic ignition, an injector and an ignition coil in front of each cylinder, using multi point injection technology, known as MPI, was used for the measurements. This ignition and injection control technology is widely used among a large number of vehicles currently in use, worldwide [41,42,43]. The measurements and measurement results reported in this publication are, thus, applicable to a large number of vehicles worldwide.

## 2. Research Methodology and Measurement Technology

The research deals with the change in engine power and the production of exhaust emissions of the measured vehicle. Practical measurements were performed in laboratory conditions. Changes in vehicle power, when the electronic component of the vehicle’s engine management was disconnected, were quantified in a cylinder power test room. An exhaust gas analyzer was used to evaluate the production of exhaust emissions.

**Figure 1:**
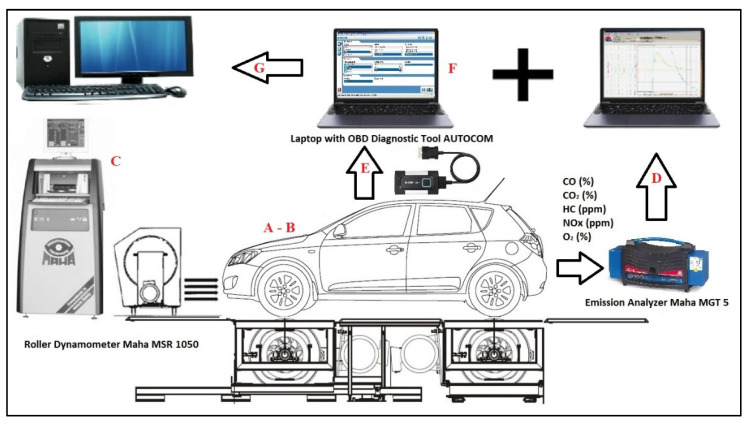
Research progress diagram (author).

The whole research process is shown in the diagram in Figure 1. The basic technical parameters of the vehicle and engine are indicated in the diagram by positions A and B. These data are contained in Table 1 Position C, D, E, and F in the diagram represent the measuring part of the research task using the measuring technique described below. After the disconnection of each electronic component, an engine power test was performed on the roller power tester (C). The results were compared with a reference measurement when no failure was simulated on the engine. Position D indicates the process for measuring exhaust emissions. The emission production was also compared with a reference measurement. We evaluated the production of emissions from several perspectives. The first was to assess the emissions of individual components of exhaust gases or whether they do not exceed a set limit. The second was to monitor the fault memory of the engine control unit (E). The memory of faults was monitored due to the ability of the vehicle to perform regular emission checks during vehicle operation. During the emission check, no fault with the designation P0XXX must be recorded in the fault memory. The last factor we observed was the display of the MIL (malfunction indicator lamp) on the vehicle’s instrument panel (F). This signals to the driver a fault detected on the vehicle engine. Between the individual tests, a driving cycle with the vehicle was performed and the fault memory of the engine control unit was checked.

All tests were performed on a Kia Ceed vehicle with the technical parameters listed in Table 1. Measurements of engine power and exhaust emissions were repeated 10 times. Extreme results have been removed from the statistics file. Appropriate statistical tools were used to evaluate representative engine power values and exhaust emissions.

The subject of the research was to monitor the change in selected operating characteristics of the vehicle (engine power, exhaust emissions) in the simulation of failure of selected electronic components that are part of engine management. The fault simulation was performed by interrupting the signal between the component and the engine control unit. For the purposes of the research, failures of the following electronic components in the form of sensors and actuators were simulated:

1. Throttle position sensor,
2. Crankshaft position sensor,
3. Oxygen sensor,
4. Suction line pressure sensor (MAP sensor—Manifold absolute pressure sensor),
5. Coolant temperature sensor,
6. Camshaft position sensor,
7. Injector,
8. Ignition coil.

When simulating a fault on the mentioned electronic components, the operation of the control unit as well as the engine itself will be negatively affected. During the implementation of individual simulations, the engine functionality, engine power, exhaust emissions, MIL indicator light and engine control unit fault memory are monitored.

Before starting the research, we checked whether there was any damage to the measuring vehicle that would invalidate the measurements and conclusions made. Automotive diagnostics and fault memory readings have confirmed that the vehicle is undamaged. A comparative measurement of power and exhaust emissions was performed, which is considered as a reference. With the results of this measurement, we further compared the results of measurements in the simulation of failures of individual electronic components.

### 2.1. Roller Dynamometer MSR 1050

Engine power is one of the basic operating characteristics of the engine, but also of the whole vehicle. Monitoring of the change in vehicle performance during the simulation of individual faults in the engine management was performed on the MAHA MSR 1050 roller dynamometer with inter-axle control. The rollers on the wheel rolls are raised above the floor plane, which improves the flow of cooling air. This ensures that we can perform long-term tests without the risk of heat build-up. The basic technical parameters of the cylindrical test bench are shown in Table 2. In addition to the measurements that have been made for research, it is possible to simulate road traffic conditions on a roller dynamometer [32].

At the roller dynamometer it is possible to perform:

Engine power measurement (continuous or discrete), load simulation (constant traction force, constant speed, driving simulation, constant engine speed), engine flexibility measurement, tachometer control.

### 2.2. Exhaust Gas Analyzer

Measuring device for purpose of emission calculation is the Maha MGT 5 exhaust gas analyser. This gas analyser is designed to measure THC (total hydrocarbon), CO (Carbon oxides), CO_2_ (carbon dioxide), O_2_ (oxygen) and NO_x_ (nitrogen oxide) emissions. The analyser operates on the principle of selective absorption which means that each component of the exhaust is assessed in the infrared range. The tested exhaust gases are conducted from a vehicle exhaust pipe to an exhaust probe that is connected to the analyser by a hose. At first, H_2_O water vapor is separated from exhaust gases, which then are led to the measuring chamber. The infrared light beam in the direction of the measuring element is weakened by the gas. The amount of attenuation of this light beam is manifested by a different wavelength depending on the type of gas. Such a method is the measured amount of THC, CO and CO_2_. On the other hand, O_2_ and NO_x_ are measured by electrochemical detection. The basic technical parameters of the analyzer are in Table 3.

## 3. Research Results

The output of individual measurements is the quantification of the degree of influence of simulated failures of the electronic components. The measured results are compared with the situation when no errors were found on the vehicle. The following section describes the extent to which the simulated electronic component failure affects the selected operating characteristics of the vehicle. The measurements were repeated 10 times. After removing the extreme values (measurements with unsuccessful implementation), the outputs from eight measurements are used for the purpose of processing the results. Average values from individual measurements were processed for the research. Electronic component failures were simulated until the necessary vehicle power and emission measurements were made.

### 3.1. Influence of Failure of Selected Electronic Components on Engine Performance

In this part of the research, it will be possible to monitor changes in engine power after the disconnection of selected electrical components. After disconnecting the component, the vehicle was tested on a roller dynamometer.

Table 4 shows the average values from the measurements. Based on the obtained data, we monitor the decrease in engine power caused by the simulation of failures of selected electronic components. As a reference measurement, the measurement performed on the vehicle without fault is used. This measurement is labeled “NOT FALUT”. Based on this, it is possible to monitor the initial changes in engine power after performing individual measurements. When observing power on wheels, we observe a percentage change in power, which depends on the change in engine power. For simplicity, we can only compare the size of the engine power loss, since the following relation applies:

(1) $$P_{engine}=P_{wheels}+P_{drag}\;\left[\mathrm{kW}\right]$$

**Figure 2:**
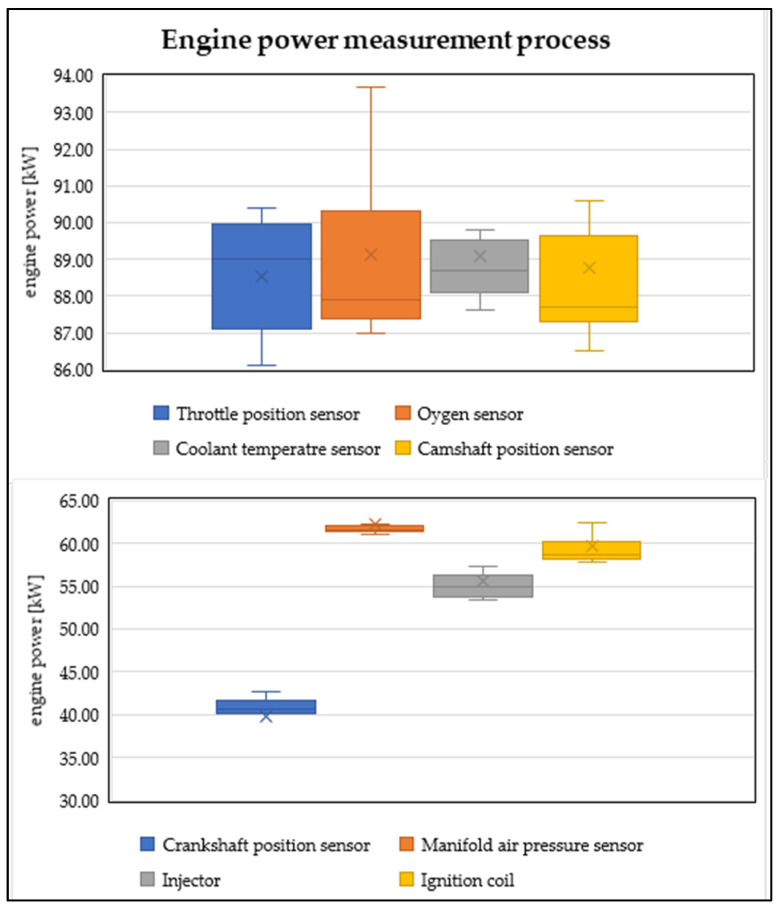
Course of power decrease in individual measurements (author).

In Figure 2, we monitor the processed outputs of power measurements in individual simulations of electronic components. The graph (Figure 2) is divided into two parts. The upper part shows the engine performance simulations of component failures that do not significantly affect performance. The lower part of the graph shows the components whose failure significantly affects engine performance. The graphs show the extreme values (maximum, minimum), average values, and lower and upper quartile.

Further, in Figure 2, it is possible to observe how the performance of the vehicle has changed during the simulation of faults on selected electronic components. The most significant decrease in engine power, in the case of sensors, occurs in the event of a fault in the crankshaft speed sensor and the MAP sensor. In the first case, the engine control unit does not have information about the crankshaft speed, but also about the detailed positions of the individual pistons in the engine cylinders. In the latter case, the control unit does not have relevant information on the amount of the intake of air. There is also a significant drop in power in the event of a fault in the injection valve and the ignition coil of one of the engine cylinders. However, these components are actuators of the engine control unit. Their malfunction, logically, significantly affects the process of fuel mixture preparation. These factors also adversely affect the safety of vehicle operations.

**Figure 3:**
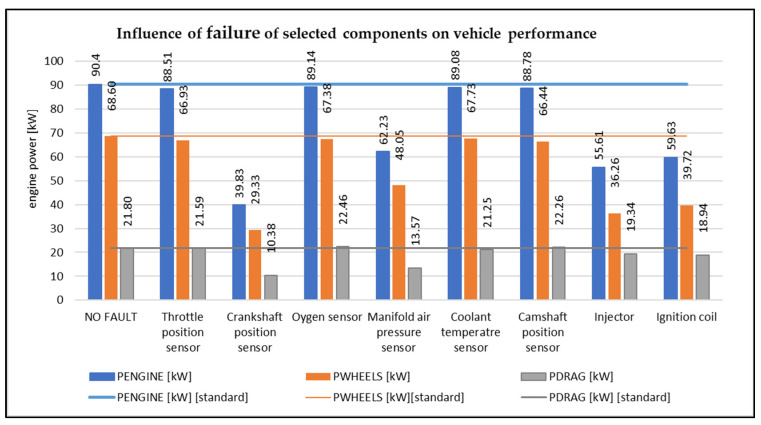
Influence of failure of selected components on vehicle performance (author).

**Figure 4:**
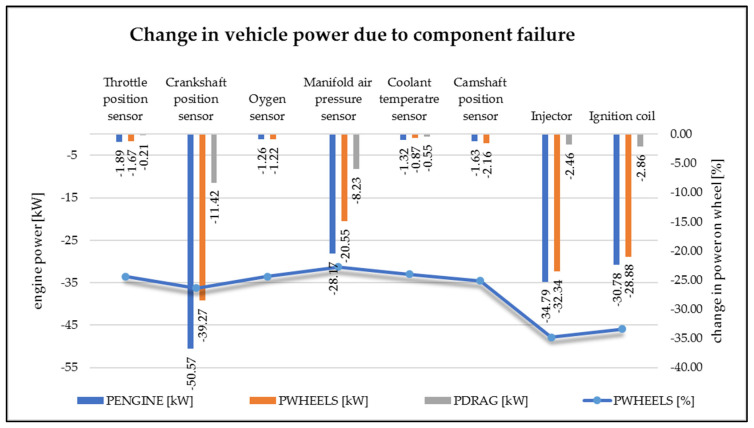
Change in engine power due to electronic component failure (author).

In Figure 3, we can see how the disconnection of individual electronic components affects the engine power. All measurements were compared when the vehicle was in a fault-free state. It can be observed that when the individual components were disconnected, there was a sudden drop in power (crankshaft speed sensor, intake manifold pressure sensor, injector, ignition coil). In the next graph (Figure 4) it is possible to observe a specific change in vehicle power when disconnecting selected components.

In the graph (Figure 4), it is possible to evaluate the amount of power loss that is caused by damage or disconnection of the component. Power loss is given in kW (kilowatt). The power loss on the wheels, the power loss of the engine, and the amount of power loss were determined.

From the graph, it is possible to observe that the biggest loss of engine power was just when the crankshaft position sensor was disconnected, namely 50.57 kW, which represents almost a 50% decrease compared to the state when the vehicle is without a fault. Another power loss was recorded on the vehicle when the intake manifold pressure sensor was disconnected (28.17 kW), the injector was disconnected (34.79 kW) and the ignition coil was disconnected (30.78 kW). Due to the disconnection of these components, the vehicle showed reduced performance. When disconnecting other monitored components, the engine did not significantly change its performance, it was comparable to the performance of the vehicle in a fault-free state. When the internal combustion engine is running, not all the power is transmitted directly to the wheels of the vehicle. Part of the vehicle’s power is loss-making. These losses are affected by several factors. The part of the power that contributes to the movement of the vehicle is the power on the wheels. Therefore, this power does not reach a value equal to the engine power. In fault simulations, it is possible to monitor how the performance on the wheels has changed. For the components that caused the loss of engine power, the part of the power that is transmitted to the wheels of the vehicle also decreased. In the graph, we see the percentage decrease in power on the wheels, which is caused by a decrease in engine power.

### 3.2. Influence of the Failure of the Selected Electronic Component on the Vehicle Emissions

As this is a vehicle with a petrol engine, it was necessary to measure exhaust emissions at both idle and high speeds. For each simulated failure, we monitored how the amount of exhaust emission production changed compared to the reference measurement.

In Table 5, we monitor the emission values for the measured vehicle. Emission production values are specified in the regulations for the specific Kia Ceed vehicle. The monitored emissions are further evaluated and compared during the next evaluation. The values in the table serve as a comparison. Exhaust emissions found in the failure simulation of selected components are compared with each other from the reference. On this principle, the authors are able to define when a vehicle produces excessive exhaust emissions

**Figure 5:**
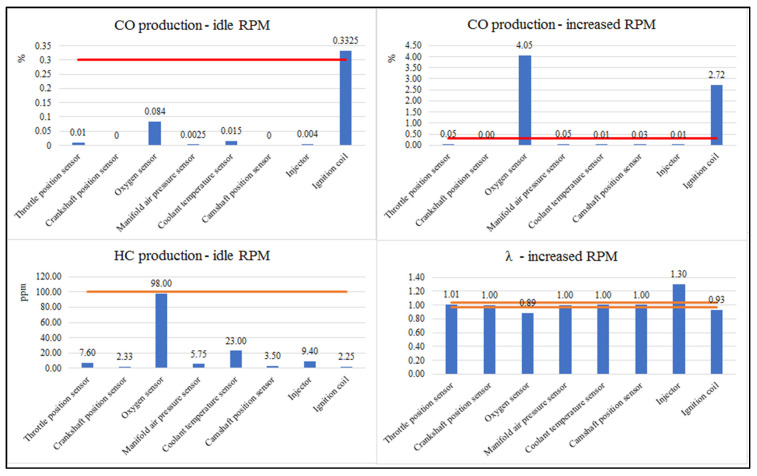
Graphic course of monitored emissions (author).

For exhaust emissions, the measurement was also repeated 10 times and extreme values were removed. The evaluation presents the average values of the individual components of the exhaust gases at increased (CO, Lambda value) and engine idling speeds (CO, HC). The lambda calculation determines the ratio between the amount of oxygen actually present in a combustion chamber vs. the amount that should have been present, to obtain perfect combustion. Figure 5 graphically shows the measured results. The graphs show the limit of the given component in a red line. In the case of CO measurement at increased engine speeds, the values were exceeded in the case of a fault simulation on the oxygen sensor and the ignition coil. At CO emissions at idle, the limit was exceeded due to ignition coil failure, and the oxygen sensor value was increased. When monitoring HC emissions at idle, they are just below the oxygen sensor failure limit. When monitoring the lambda value, the increased value is monitored in the event of an injector fault, and on the contrary, in the event of an oxygen sensor fault, the value is lower than the overwritten limit.

Table 6 is a final evaluation of the measured components of the exhaust gases. During the implementation of the emission control in the Slovak Republic, “additional parameters” are further evaluated. These were not further evaluated in the research. We focused only on the basic parameters that fundamentally affect the result of emission control.

For simplification and correct understanding of the interpreted results, the authors prepared a final table (Table 6), where it is possible to monitor the identified indicators. In the event of individual sensor failures, it is possible to monitor whether the detected value of exhaust emissions complies with the specified regulations. At the oxygen sensor, we monitor the increased production of CO. This production is also increased in the event of an ignition coil and injector failure.

It is clear from Table 7 to what extent the vehicle will be affected due to the failure of the electronic component. Some failures of selected components have no or minimal effects on vehicle operation. This means that the vehicle’s performance remains unchanged, the exhaust emissions remain within the limit and the driver is not informed of the fault (control MIL is off). Such components are the oxygen sensor and the throttle position sensor. The driver may not be aware of their fault until the vehicle is connected for diagnostics, where an error code is displayed. In Table 8, you can see the explanation for the color change shown in Table 7.

Other components are those whose failure manifests itself as soon as they occur. It is a coolant temperature sensor and a camshaft speed sensor. In the event of a temperature sensor fault, the fan on the motor starts immediately at maximum power. This is because the control unit does not receive this information from the sensor and, thus, prevents damage to the motor by overheating. With the crankshaft speed sensor, the engine speed (rpm) is no longer displayed to the driver. It will reappear after a short time. The engine control unit calculated them using other sensors. The driver does not receive information about the failure of these components via the MIL control panel. The emission of the vehicle, as well as its performance, remains similar to the state when no failure occurs on the vehicle.

Another group is components of which the driver detects a failure in the loss of vehicle power (crankshaft position sensor, intake manifold pressure sensor, injector, ignition coil). If these components fail, the vehicle loses performance and there is also a problem with the Emission Inspection. If the intake manifold pressure sensor fails, the vehicle loses engine power (lower by 28.17 kW), but the vehicle’s emissions are OK and the driver is not informed of the fault. The most serious faults are faults in the crankshaft position sensor, injector, and ignition coil. Due to the failure of these components, the vehicle loses power and also increases the production of exhaust emissions, above the permissible limit. When simulating a crankshaft position sensor failure, engine emissions are OK but power is reduced by almost 50 kW. This is because the control unit is operating in emergency mode and will not allow the engine to reach higher speeds. In the event of its failure, the vehicle has a problem maintaining a stable engine speed within the prescribed range for a specified interval. The driver does not receive information about this fault either (the MIL indicator is off). The only faults where the driver is informed via the MIL are the ignition coil fault and the injector fault. In the event of these faults, the MIL indicator light on the instrument panel illuminates and flashes. These are faults that are indicated in accordance with the approved regulations.

From the point of view of performing the emission inspection in the conditions of the Slovak Republic, the vehicle would not be assessed as eligible for any of these simulated faults, as an error in the form P0xxx is written in the fault memory. For this reason, too, it can be stated that the vehicle operator should make sure during each vehicle maintenance that a fault is recorded in the fault memory, which is related to the operation of the vehicle and also to the excessive production of exhaust emissions.

## 4. Discussion

Research is specified to quantify data on engine power and exhaust emissions in cases where the electronic engine management may have different failures. This describes how the simulation of failures of selected components affects the operation of the vehicle. It points to emission values, such as air pollutants [45], that are produced by a vehicle in the event of various failures. According to some studies [46,47,48], the way in which these values are measured is important. Studies point to the fact that emissions can also be affected by the method of measurement. Skrúcaný et al. proved this in research, when he monitored the fuel consumption of a road vehicle [49]. It is clear from the research that fuel consumption, as well as emission production, were different when measured in the laboratory and in real driving. For this reason, vehicle emissions were measured in the research, according to the valid methodological instructions by which emission controls are performed in the Slovak Republic. Mileage also affects the vehicle’s emissions. Research has confirmed that as the number of kilometers traveled increases, the overall level of emissions produced will increase [50]. In our case, however, it is not a question of monitoring long-term emission developments. However, in the future, such a measurement can be made, and this claim confirmed [51]. In the event of a failure of the injector and the ignition coil, the prescribed limits were exceeded, in particular, CO emissions. This exceedance may not be perceived by the vehicle operator, but overall, it has a negative impact on the environment. This is confirmed by the fact that the correct functioning of the electronic components of the vehicle’s engine management contributes to the production of emissions that do not exceed the limits during the emission control. Castillo et al. reached similar conclusions in their research. They found out how the production of vehicle emissions changes after disconnecting the ignition coil. They found an increase in CO production when the ignition coil failed. The production value was 5.21%, while the research also simulated an air filter failure. The results obtained are comparable, as the Kia Ceed produced CO 2.72%, but the air filter was flawless [36]. Greene et al., in their research, proved that the production of emissions for the road transport sector decreases year on year. One of the reasons is precisely the design of internal combustion engines in new vehicles, as well as the stricter limits that new vehicles must meet [52]. Johnson also describes developments in vehicle emission control in his study. It is also working on proposing new emission limits that are comparable to Japanese standards. This is a follow-up to the authors’ research because the limits for emission controls are also lower [53].

A large number of studies have dealt with the change in engine power due to the fuel used [54,55,56]. An important part of the research is the evaluation of engine power, which was measured in the failure simulation of a selected component. The observed power drop was recorded in the event of a fault in the crankshaft position sensor, MAP sensor, injector and also, ignition coil. The largest decrease was recorded when the crankshaft position sensor was disconnected, namely 50.57 kW. Azzoni et al. conducted similar research, where they were able to diagnose an engine failure using a crankshaft speed sensor. The research was based on monitoring crankshaft speed fluctuations [57]. Seifi et al. investigated the change in engine power after adding water to the fuel. After adding water to the fuel, the engine power and torque increased. From the point of view of power measurement, these studies are similar, as the engine power has been monitored, which has changed due to the environment [30]. The result of the component failure simulation is that the vehicle will be able to operate, but its properties will not be comparable to the vehicle in a fault-free state. There are several ways to monitor the impact of a component failure. This is evidenced by the research of Organ et al., which processed the impact of the vehicle failure on its operation. The research looked at the impact of damage per kilometer of the route traveled by the vehicle. The results of the study showed a significant increase in THC and CO of up to 317% (0.604 g/km) and 782% (5.351 g/km), in the case of a simulated high voltage failure at the oxygen sensor and the sticky control valve. The largest increase was in NO and exhaust systems. When simulated in the laboratory, disconnecting the oxygen sensor increased CO emissions by almost 800%, which is approximately the same value that the vehicle reached in operation. This comparison clearly points to the importance of properly maintaining emissions and engine hardware systems, in order to achieve optimal fuel consumption and satisfactory emission levels that could be reproduced in other regions with prescribed emission control [58]. The drop in power caused by a component failure is noticeable and the driver registers it. The decrease in performance will not only worsen the driving characteristics of the vehicle, but there is a presumption that the production of exhaust emissions will increase. Another important piece of information is the fault memory of the engine control unit. Every vehicle with such a fault has an error code written in the fault memory. For this reason, this fault memory is checked during regular emission inspections.

## 5. Conclusions

In the evaluation of the research, we came to the conclusion that the damage or malfunction of selected electronic components of the engine management has a significant impact on the operation of the vehicle. Each malfunction manifests itself in a different way. We observed how the damage to selected components affects the engine power and also the production of exhaust emissions. The researched components can be divided into categories.

The first group is those components whose damage does not result in any changes on the vehicle (MIL indicator does not illuminate). It is an oxygen sensor and a throttle position sensor. By damaging these components, engine power and emissions are comparable to a state of failure. However, with a non-functional oxygen sensor, there has been a significant increase in the production of CO and HC, components that are toxic and carcinogenic to humans. The danger is also the fact that the MIL indicator has not been activated and the engine power has not been significantly reduced. The vehicle would, thus, probably continue to operate with excessive emissions without the driver’s knowledge of this condition, until a regular emission inspection has been carried out. As the vehicle’s engine ran on a rich mixture, as shown in Figure 5, over time, the catalyst efficiency is likely to decrease due to unburned hydrocarbon pollution and a further increase in harmful emissions.

Another group is components where the monitored indicators (engine power and emission production) do not change, but the driver can detect their failure in another way [59]. Such behavior has been detected with the coolant temperature sensor and the camshaft position sensor. If these components fail, the MIL indicator on the driver’s instrument panel will not illuminate. This means that he is not informed about their failure.

The third group consists of components whose failure results in a loss of engine power. This is also evidenced by related research, in which the diagnosis of the same components resulted in results that were comparable to the results of our research [60,61]. We have included in this group components, such as crankshaft position sensor, intake manifold pressure sensor, injector, ignition coil. By simulating their failure, engine power was lost. In the event of an injection and ignition coil failure, we can observe an increased production of exhaust emissions. They are also the only components whose fault is signaled to the driver via an indicator light (MIL indicator-on, flashing). The vehicle’s engine was running on a poor lean injector. In the case of further driving with such a vehicle, the exhaust valve on the given cylinder would probably be damaged and, consequently, the catalytic converter would be gradually destroyed. The reason would be the fact that the lean mixture burns longer and, thus, causes high thermal stress within the components. The catalytic converter is one of the most expensive spare parts for the vehicle due to its precious metal content.

The conclusions of the research represent important findings from the environment of engine management and fuel mixture preparation. At the same time, they point out the influence of individual electronic components in the internal combustion engine on engine performance and regulation of exhaust emissions. By functioning properly, we can prevent the excessive production of exhaust emissions into the air. The proper functioning of the components, and the vehicle as a whole, contributes to the overall safety on the road.

Research has shown that each failure affects the operation of the vehicle and also the safety of operation. At least only because the fault appears as a code, written in the engine fault memory. This means that at the time of the emission inspection, the vehicle would be assessed as unfit during the emission inspection for any damage. It is possible to state and recommend vehicle inspection by car diagnostics at each service operation. As research has confirmed to us that the driver does not have to have any information about the damage, any damage can lead to the vehicle producing emissions that exceed the specified values and contribute to excessive environmental pollution. Based on the findings of this publication, it can be strongly recommended that the requirement for such an adjustment of engine control units be included in the drafting of the relevant legislation, in which, in the event of a fault with serious consequences, such as an oxygen sensor malfunction, the MIL would be activated immediately and, after a certain time, the engine power would be gradually reduced, and the driver would be forced to rectify the fault.

The findings of this publication can also be taken into account when modeling the quantity of emissions produced by road transport.

## Figures and Tables

**Table 1:** Technical parameters of Kia Ceed (data processed by the author).

Technical Parameters of the Measured Vehicle	
Brand	KIA
Trade name	Cee’d
Engine code	G4FC
Number of cylinders	4
Cylinder displacement	1591 cm^3^
Highest engine power	90.00 kW
Speed at max. moment	6200 min^−1^
Highest design speed	192 km·h^−1^
Fuel type	Petrol
Length	4265 mm
Width	1790 mm
Height	1480 mm
Operating weight	1163 kg
Maximum permissible total weight	1710 kg

**Table 2:** Technical data MAHA MSR 1050 [32].

Technical Data MAHA MSR 1050	
Rollers diameter	762 mm
Number of electric eddy-current brakes	3
Number of electric engines	2
Wheels gauge (min.–max.)	900–2300 mm
Measurable wheelbase	1900–3500 mm
Permissible axle load	2500 kg
Top speed	320 km/h
Rotational roller unit weight	700 kg
Front axle wheel performance	700 kW
Rear axle wheel performance	1400 kW
Front axle tractive force	8600 N
Rear axle tractive force	17,200 N

**Table 3:** Technical data MAHA MGT 5 [44].

Technical Data MAHA MGT 5					
measured gases	CO	CO_2_	HC	O_2_	NO_x_
measuring ranges	0–15.00 Vol%	0–20.00 Vol%	0–2000 ppm Vol (Hexan) 0–4000 ppm Vol (Propan)	0–25.00 Vol%	0–5000 ppm Vol
the accurracy of measuring	0.06 Vol%	0.5 Vol%	12 ppm	0.1 Vol%	32–120 ppm Vol
measurement principle	infrared	infrared	infrared	electro-chemical	electro-chemical
resolution of values	0.001	0.01	0.1	0.01	1
measuring range deviation	less than ± 0.6% of the final value of the measuring range				
flow	max. 3.5 L/min • min 1.5 L/min				
gas outlet	approx 2.5 L/min				
condensate drain	automatically, continuously • approx. 1 L/min				
working pressure	750–1100 mbar				
pressure fluctuations	max. error 0.2% with fluctuations of 5 kPa				

**Table 4:** Average values recorded during power measurement (author).

Monitored Values	$P_{ENGINE}$	$P_{WHEELS}$		$P_{DRAG}$
	[kW]	[kW]	[%]	[kW]
Not Falut	90.4	68.60	75.88	21.80
Throttle position sensor	88.51	66.93	75.61	21.59
Crankshaft position sensor	39.83	29.33	73.65	10.38
Oygen sensor	89.14	67.38	75.59	22.46
Manifold air pressure sensor	62.23	48.05	77.22	13.57
Coolant temperatre sensor	89.08	67.73	76.02	21.25
Camshaft position sensor	88.78	66.44	74.84	22.26
Injector	55.61	36.26	65.21	19.34
Ignition coil	59.63	39.72	66.62	18.94

**Table 5:** Emission values for the monitored vehicle (author).

Engine speed [rpm]	Idle RPM		Increased RPM	
	650–850		2200–2400	
Monitored emissions	CO [%]	HC [ppm]	CO [%]	λ
	to 0.3	to 100	to 0.3	0.97–1.03

**Table 6:** Emissions measured during disconnection of monitored components (author).

Monitored Values	Average Values			
	Idle RPM		Increased RPM	
	CO	HC	CO	λ
Throttle position sensor	OK	OK	OK	OK
Crankshaft position sensor	OK	OK	OK	OK
Oxygen sensor	OK	OK	over limit	low limit
Manifold air pressure sensor	OK	OK	OK	OK
Coolant temperature sensor	OK	OK	OK	OK
Camshaft position sensor	OK	OK	OK	OK
Injector	OK	OK	OK	over limit
Ignition coil	over limit	OK	over limit	low limit

**Table 7:** Influence of component disconnection on monitored indicators (author).

Monitored Values	MIL Indicator	Fault Memory Error	Emission Production	Result of Emission Inspection	Power Measurement Result
NO FAULT	off	without record	in the standard	eligible	without loss of power
Throttle position sensor	off	P0123	in the standard	ineligible	without loss of power
Crankshaft position sensor	off	P0339	in the standard	ineligible	power loss
Oxygen sensor	off	P0030; P0134	out of standard	ineligible	without loss of power
Manifold air pressure sensor	off	P0108	in the standard	ineligible	power loss
Coolant temperature sensor	off	P0118	in the standard	ineligible	without loss of power
Camshaft position sensor	off	P0343	in the standard	ineligible	without loss of power
Injector	lights/flashes	P0201	out of standard	ineligible	power loss
Ignition coil	lights/flashes	P0300; P0301	out of standard	ineligible	power loss

**Table 8:** Explanation of terms from Table 7 (author).

	the vehicle shows increased emissions—above the set limit
	When measured on a roller dynamometer, the vehicle loses power due to the disconnection of selected components
	the driver is informed by the engine control unit of the fault

## Data Availability

Not applicable.

## References

[1] Dillender M. (2021). Climate Change and Occupational Health Are There Limits to Our Ability to Adapt?. J. Hum. Resour..

[2] Coelho S., Rafael S., Lopes D., Miranda A., Ferreira J. (2021). How changing climate may influence air pollution control strategies for 2030?. Sci. Total Environ..

[3] Caban J. (2021). Traffic congestion level in 10 selected cities of Poland. Sci. J. Sil. Univ. Technol. Ser. Transp..

[4] Cohen A.J., Anderson H.R., Ostro B., Pandey K.D., Krzyzanowski M., Künzli N., Gutschmidt K., Pope A., Romieu I., Samet J.M. (2005). The Global Burden of Disease Due to Outdoor Air Pollution. J. Toxicol. Environ. Health Part A.

[5] Liu Y., Ao C. (2021). Effect of air pollution on health care expenditure: Evidence from respiratory diseases. Health Econ..

[6] Kelly F.J., Fussell J.C. (2011). Air pollution and airway disease. Clin. Exp. Allergy.

[7] Harrison R.M., Beddows D.C. (2017). Efficacy of Recent Emissions Controls on Road Vehicles in Europe and Implications for Public Health. Sci. Rep..

[8] Johnson T.V. (2009). Diesel emission control in review. SAE Int. J. Fuels Lubr..

[9] Reşitoğlu İ.A., Altinişik K., Keskin A. (2015). The pollutant emissions from diesel-engine vehicles and exhaust after-treatment systems. Clean Technol. Environ. Policy.

[10] Neeft J.P., Makkee M., Moulijn J.A. (1996). Diesel particulate emission control. Fuel Process. Technol..

[11] Jeanneret B., Buttes A.G.D., Pelluet J., Keromnes A., Pélissier S., Le Moyne L. (2021). Optimal Control of a Spark Ignition Engine Including Cold Start Operations for Consumption/Emissions Compromises. Appl. Sci..

[12] Doolan R., Muntean G.M. (2016). EcoTrec—A novel VANET-based approach to reducing vehicle emissions. IEEE Trans. Intell. Transp. Syst..

[13] Beňová D., Settey T., Slávik R., Gnap J. (2019). Examination of possibilities of air quality in-creasing in cities using freight vehicles with an ecology type of drive. Perner’s Contacts.

[14] Mahdinia I., Arvin R., Khattak A.J., Ghiasi A. (2020). Safety, Energy, and Emissions Impacts of Adaptive Cruise Control and Cooperative Adaptive Cruise Control. Transp. Res. Rec. J. Transp. Res. Board.

[15] Kulkarni P., Rajani P., Varma K. (2016). Development of On Board Diagnostics (OBD) testing tool to scan emission control system. Proceedings of the 2016 International Conference on Computing Communication Control and Automation (ICCUBEA).

[16] Naik P., Kumbi A., Telkar N., Kotin K., Katti K.C. (2017). An automotive diagnostics, fuel efficiency and emission monitoring system using CAN. Proceedings of the 2017 International Conference on Big Data, IoT and Data Science (BID).

[17] Niazi M.A.K., Nayyar A., Raza A., Awan A.U., Ali M.H., Rashid N., Iqbal J. (2013). Development of an On-Board Diagnostic (OBD) kit for troubleshooting of compliant vehicles. Proceedings of the 2013 IEEE 9th International Conference on Emerging Technologies (ICET).

[18] OBD Diagnostics. https://www.autodiagnostika.jantolak.sk/?%C8l%E1nok-5-%DAvod-do-diagnostiky-obd-obd2-eobd-zariadenia,259.

[19] Emission System Fault Diagnosis Part 1. http://www.turbo-tec.eu/cz/blog/aplikacia-vysledkov-emisnej-kontroly-na-diagnostika-poruch-emisneho-systemu-motorov-cast-1/.

[20] Emission System Fault Diagnosis Part 2. http://www.turbo-tec.eu/cz/blog/aplikacia-vysledkov-emisnej-kontroly-na-diagnostika-poruch-emisneho-systemu-motorov-cast-2/.

[21] Hickman A. (1994). Vehicle maintenance and exhaust emissions. Sci. Total Environ..

[22] Díaz S., Bernard M.R., Bernard Y., Bieker G., Lee K., Mock P., Mulholland E., Ragon P.-L., Rodriguez F., Tietge U. European Vehicle Market Statistics 2021/2022. https://theicct.org/publication/european-vehicle-market-statistics-2021-2022/.

[23] Held M., Rosat N., Georges G., Pengg H., Boulouchos K. (2021). Lifespans of passenger cars in Europe: Empirical modelling of fleet turnover dynamics. Eur. Transp. Res. Rev..

[24] Hudec J., Šarkan B., Cződörová R. (2021). Examination of the results of the vehicles technical inspections in relation to the average age of vehicles in selected EU states. Transp. Res. Procedia.

[25] Binar T., Svarc J., Rozlivka J. Assesment of properties affecting the lyfe cycle of the spare part of the means of transport. Proceedings of the International Conference on Traffic and Transport Engineering (ICTTE).

[26] Methodical Instruction of the Emission Inspection. https://seka.sk/storage/app/media/stranky/legislativa/metodiky/2021/MP_2_2020%20-%20pln%C3%A9%20znenie.pdf.

[27] Zheng X., Lu S., Yang L., Yan M., Xu G., Wu X., Fu L., Wu Y. (2020). Real-world fuel consumption of light-duty passenger vehicles using on-board diagnostic (OBD) systems. Front. Environ. Sci. Eng..

[28] Kuranc A. (2015). Exhaust emission test performance with the use of the signal from air flow meter. Eksploat. I Niezawodn..

[29] Dynamometer Power Measurement. http://www.profituning.sk/meranie-vykonu-na-valcovej-stolici-maha-lps3000/.

[30] Seifi M.R., Hassan-Beygi S.R., Ghobadian B., Desideri U., Antonelli M. (2016). Experimental investigation of a diesel engine power, torque and noise emission using water–diesel emulsions. Fuel.

[31] Wiśniowski P.K., Slezak M., Niewczas A. (2019). Simulation of road traffic conditions on a chassis dynamometer. Arch. Motoryz..

[32] Manual Maha MSR 1050. https://www.manualslib.com/manual/1578646/Maha-Powerdyno-Msr-Series.html#page=2-manual.

[33] Rizzoni G., Ribbens W.B. (1989). Crankshaft position measurement for engine testing, control, and diagnosis. Proceedings of the IEEE 39th Vehicular Technology Conference.

[34] Jian X., Zhang C., Wang X., Wang P. (2018). Simulation test on failures of an electronically controlled gasoline engine. J. Chang. Univ. (Nat. Sci. Ed.).

[35] Dong Q., Yuan H., Jian X., Li Y., Jiao S. (2013). Fault simulation test of oxygen sensor for natural gas engine. J. Traffic Transp. Eng..

[36] Castillo-Calderón J., Sinche D.D., Jaura R.C., Campoverde D.R. (2021). Influence of Failures not Detected by the On-Board Diagnostic System on the Performance and Pollutant Emissions of a Spark Ignition Engine. Adv. Intell. Syst. Comput..

[37] Wang Y., Zhang F., Cui T., Zho J. (2016). Fault diagnosis for manifold absolute pressure sensor(MAP) of diesel engine based on Elman neural network observer. Chin. J. Mech. Eng..

[38] Kamalakar S.S., Vanjale M.S. Notifying and inspecting vehicle emission and temperature of vehicle engine. Proceedings of the 2017 Conference on Emerging Devices and Smart Systems (ICEDSS).

[39] Liu L., Li J., Wang L., Zhao W., Qin H., Wang Y. (2019). Experimental Study on Effects of OBD II Diagnostics on of Emissions for Light Vehicles. Adv. Intell. Syst. Comput..

[40] Huang Y., Ng E.C., Yam Y.-S., Lee C.K., Surawski N., Mok W.-C., Organ B., Zhou J.L., Chan E.F. (2019). Impact of potential engine malfunctions on fuel consumption and gaseous emissions of a Euro VI diesel truck. Energy Convers. Manag..

[41] European Vehicle Market Statistics, Pocketbook 2020/2021. https://theicct.org/sites/default/files/publications/ICCT_EU_Pocketbook_2020_Web_Dec2020.pdf.

[42] Highlights of the Automotive Trends Report, Automotive Trends Report. https://www.epa.gov/automotive-trends/highlights-automotive-trends-report.

[43] ASEAN Fuel Economy Roadmap for the Transport Sector 2018–2025: With Focus on Light-Duty Vehicles. https://asean.org/wp-content/uploads/2021/11/ASEAN-Fuel-Economy-Roadmap-FINAL.pdf.

[44] Manual Maha MGT 5. https://www.maha-india.in/cps/rde/xbcr/SID-7A029843-07D1D0F8/maha_de/BRO_MAHA_alle_Abgastester_EN.pdf.

[45] Buckeridge D.L., Glazier R., Harvey B.J., Escobar M., Amrhein C., Frank J.W. (2002). Effect of motor vehicle emissions on respiratory health in an urban area. Environ. Health Perspect..

[46] Zhang J., Peng J., Song C., Ma C., Men Z., Wu J., Wu L., Wang T., Zhang X., Tao S. (2020). Vehicular non-exhaust particulate emissions in Chinese megacities: Source profiles, real-world emission factors, and inventories. Environ. Pollut..

[47] Perry R., Gee I. (1995). Vehicle emissions in relation to fuel composition. Sci. Total Environ..

[48] Fontaras G., Zacharof N.-G., Ciuffo B. (2017). Fuel consumption and CO_2_ emissions from passenger cars in Europe—Laboratory versus real-world emissions. Prog. Energy Combust. Sci..

[49] Skrúcaný T., Stopková M., Stopka O., Kalašová A., Ovčiarik P. (2020). User’s determination of a proper method for quantifying fuel consumption of a passenger car with compression ignition engine in specific operation conditions. Open Eng..

[50] Zhang Q., Fan J., Yang W., Ying F., Bao Z., Sheng Y., Lin C., Chen X. (2018). Influences of accumulated mileage and technological changes on emissions of regulated pollutants from gasoline passenger vehicles. J. Environ. Sci..

[51] Faiz A., Weaver C.S., Walsh M.P. (1996). Air Pollution from Motor Vehicles: Standards and Technologies for Control-Ling Emissions.

[52] Greene D.L., Baker H.H., Plotkin S.E. (2010). Reducing Greenhouse Gas Emissions from US Transportation.

[53] Johnson T.V. (2008). Diesel Emission Control in Review. SAE Int. J. Fuels Lubr..

[54] Al-Hasan M. (2003). Effect of ethanol–unleaded gasoline blends on engine performance and exhaust emission. Energy Convers. Manag..

[55] Campos-Fernandez J., Arnal J.M., Gomez J., Lacalle N., Dorado M.P. (2013). Performance tests of a diesel engine fueled with pentanol/diesel fuel blends. Fuel.

[56] Forson F., Oduro E., Hammond-Donkoh E. (2004). Výkon zmesi jatrofy sa spája so vznetovým motorom. Obnov. Energ..

[57] Azzoni P., Cantoni G., Minelli G., Moro D., Rizzoni G., Ceccarani M., Mazzetti S. (1995). Measurement of Engine Misfire in the Lamborghini 533 V-12 Engine Using Crankshaft Speed Fluctuations. SAE Trans..

[58] Organ B., Huang Y., Zhou J.L., Yam Y.-S., Mok W.-C., Chan E.F. (2020). Simulation of engine faults and their impact on emissions and vehicle performance for a liquefied petroleum gas taxi. Sci. Total Environ..

[59] Adaileh W.M. (2013). Engine fault diagnosis using acoustic signals. Applied Mechanics and Materials.

[60] Toma M., Micu D., Andreescu C. (2019). Influences of engine faults on pollutant emission. Procedia Manuf..

[61] Zareei J., Kakaee A.H. (2013). Study and the effects of ignition timing on gasoline engine performance and emissions. Eur. Transp. Res. Rev..

